# Potential of Salivary Biomarkers in Autism Research: A Systematic Review

**DOI:** 10.3390/ijms221910873

**Published:** 2021-10-08

**Authors:** Katarína Janšáková, Klaudia Kyselicová, Daniela Ostatníková, Gabriela Repiská

**Affiliations:** Institute of Physiology, Faculty of Medicine, Comenius University in Bratislava, 81372 Bratislava, Slovakia; klaudia.kyselicova@fmed.uniba.sk (K.K.); daniela.ostatnikova@fmed.uniba.sk (D.O.); gabriela.repiska@fmed.uniba.sk (G.R.)

**Keywords:** autism, biomarkers, saliva, saliva collection, saliva limitations

## Abstract

The diagnostic process for autism spectrum disorders (ASD) is based on a behavioral analysis of the suspected individual. Despite intensive research, no specific and valid biomarker has been identified for ASD, but saliva, with its advantages such as non-invasive collection, could serve as a suitable alternative to other body fluids. As a source of nucleic acid of both human and microbial origin, protein and non-protein molecules, saliva offers a complex view on the current state of the organism. Additionally, the use of salivary markers seems to be less complicated not only for ASD screening but also for revealing the etiopathogenesis of ASD, since enrolling neurotypical counterparts willing to participate in studies may be more feasible. The aim of the presented review is to provide an overview of the current research performed on saliva in relation to ASD, mutual complementing, and discrepancies that result in difficulties applying the observed markers in clinical practice. We emphasize the methodological limitations of saliva collection and processing as well as the lack of information regarding ASD diagnosis, which is critically discussed.

## 1. Introduction

Autism spectrum disorders (ASD) represent a group of neurodevelopmental disorders including autism disorder, Asperger syndrome, childhood disintegrative disorder, and pervasive developmental disorder not otherwise specified [1]. The prevalence of ASD is rising considerably worldwide with a higher prevalence in male than in female patients, with an estimated ratio of 4:1 [2]. Several factors contributing to ASD development are considered relevant, and key roles are played by genetics, environmental factors, and parental health status/health status of the mother [3,4]. As a consequence of these factors, several candidate genes, polymorphism, and/or the related disbalance of neuropeptides and steroid hormones have been defined as related to ASD pathogenesis [5,6]. Meta-analyses comprising studies in different geographical locations since 1966, until ten years ago [7] indicated a global incidence of ASD of between 0.6% and 0.7% [7,8]. Over the past decade, the reported global prevalence has increased markedly [8,9]. Studies suggest that part of this increase is attributable to changes in diagnostic criteria, an increased awareness, and the ability to diagnose at a younger age [7,10]. The current prevalence of ASD observed by the Early Autism and Developmental Disabilities Monitoring (Early ADDM) Network in the United States during the surveillance years 2010, 2012, and 2014 is estimated at 3% [11]. Unfortunately, there are no complex surveillance activities in most countries of the world; therefore, the actual global prevalence of ASD still remains somewhat unclear.

Currently, ASDs are diagnosed using a solely psychological approach, but a biological marker suitable for diagnostics is still missing despite intensive research and a presence of several indices related to biological assessment. Diagnostics are performed according to the Statistical Manual of Mental Disorders 5th edition (DSM-5) [12]. Suspected individuals undergo a diagnostic process based on the Autism Diagnostic Observation Schedule 2nd revision (ADOS-2) and Autism Diagnostic Interview-Revised (ADI-R) [13,14]. Both of these diagnostic tools are internationally considered as the gold standard and are based on the observation of behavioral symptoms of the individual and an interview with the parent or caregiver of the corresponding individual.

The background of ASD etiopathogenesis is multifactorial, consisting of the genetics, environment, and parental age and health status [15]. Since the specific etiology of ASD remains dominantly unknown, discovering a tangible biomarker is challenging. The global scientific community is looking for these markers predominantly in blood, amniotic fluid, or alternatively in saliva [16,17,18]. Saliva presents an underrated body fluid with many advantages and abilities. Its number of detectable markers is comparable to those found in blood, and despite the lower concentration of these markers, saliva might be a useful alternative to blood [19]. The collection of saliva is non-invasive, easy, may be performed repeatedly during a relatively short period, and thus, trained staff is not needed. The monitoring of markers obtained in saliva could not only serve diagnostic purposes but also for monitoring the course of diseases [20]. Previous studies pointed to the usability of saliva in researching a wide spectrum of diseases, but in the field of neurological disorders, and especially autism, saliva is rarely considered.

The aim of this review is to summarize the current ASD research devoted to biomarkers in saliva. Moreover, a general overview regarding salivary markers, detection methods, and the limitation of saliva use is provided as well.

## 2. Methods

**Figure 1:**
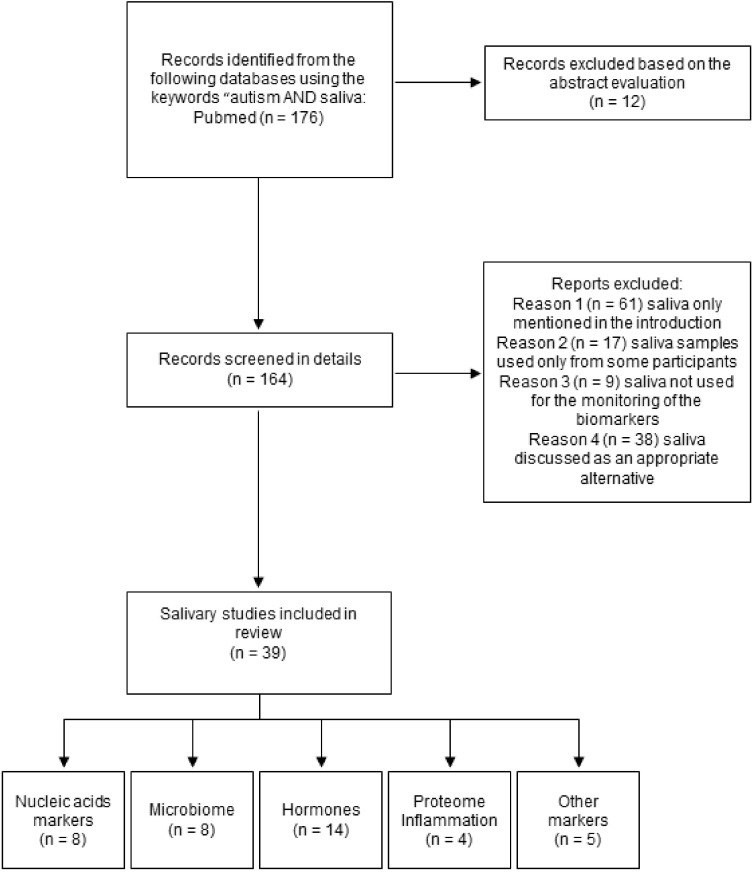
Flow diagram and research strategy for this review.

Three authors searched for these studies independently and the inclusion criteria were set for articles published in the English language since 2005 until the first half of 2021. All publications were searched via the PubMed database using the following keywords: “saliva AND autism“ or “salivary biomarkers in autism“. A total of 176 papers were found that were further processed based on the scope and purpose of this review (Figure 1) [21]. Thus, further processing of the obtained number of papers included searching for more specific terms such as “salivary profile and autism”, “salivary microbiome AND autism”, “salivary nucleic acids in autism”, “saliva AND DNA markers AND autism”, “saliva AND RNA markers AND autism”, “salivary hormones AND autism”, “monitoring of saliva in autism”, saliva AND autism research, “ proteome AND saliva AND autism”, “saliva AND inflammation AND autism”, “oxidative stress in saliva AND autism”, “salivary ions AND autism”, “non-protein salivary biomarkers AND autism”, and other modifications of these formulations. Studies monitoring (a) all possible salivary biomarkers or salivary indicators in individuals diagnosed with autism spectrum disorder and healthy controls or (b) biomarkers and parameters measured over time in saliva of individuals with autism spectrum disorder were evaluated as eligible, and thus, included in the final selection. The texts of the relevant studies were examined, and all the important data were extracted and used for the construction of tables according to the specific core topics, which were as follows: nucleic acids, microbiome, hormones, inflammation, proteome, oxidative stress, and other markers and parameters. Regarding the participants involved in the studies, an established autism diagnosis and/or healthy control was substantial. The important information presented in the selected included studies was (a) saliva collection process (e.g., unstimulated vs. stimulated saliva, duration of salivation), (b) timing of the collection process, (c) handling of the saliva samples (e.g., centrifugation), (d) the extraction procedure for the analysis of the nucleic acids, (e) detection and analytical methods for the analysis of individual biomarkers, and (f) sample size. Studies investigating various markers in plasma or serum, intervention studies, as well as animal studies or those performed on cell lines were not considered for the purpose of this review. Other articles, such as review articles and/or case reports/non-saliva original articles, were used for the completion of individual sections.

## 3. Challenges in Salivary Biomarker Research

Saliva is a suitable source of a wide spectrum of markers detectable by using different methods. Predominantly, salivary research aims to find a stable marker facilitating a diagnostic process or finding new biomarkers related to specific diseases [20]. For this purpose, saliva has been applied in the fields of oral and systemic diseases [22,23,24].

As mentioned above, saliva has several advantages in comparison to other biofluids. One of them is a patient-friendly collection process. Principally, there are two ways to collect saliva. First, the collection of unstimulated whole mouth saliva is performed by passive drooling of saliva into the mouth and then the entire mouth content is released into a sterile tube. Second, the production of saliva is stimulated using various stimulants such as a cotton swab, citric acid, chewing paraffin block, or filter paper, etc. [25]. Both processes have some advantages as well as disadvantages. Unstimulated saliva might be difficult to collect in some cases, for instance, from little children, elderly individuals, or those with affected salivary glands [26,27,28]. In contrast, stimulated saliva offers a higher volume of saliva obtained in a shorter time, which makes this collection process more comfortable and simpler [25,29]. However, the volume of saliva obtained by different stimulating collection techniques varies [30]. Additionally, several studies have reported differences in the salivary profile of stimulated saliva compared to unstimulated saliva [31,32]. Stimulation affects the snapshot of salivary proteome and, thus, affects the number of proteins [33]. Therefore, it is questionable how the results of studies using different collection approaches are comparable. Discrepancies such as these complicate summarizing the acquired data, especially in fields of research where saliva is not commonly used. The combination of various collection techniques within a project might lead to heterogenous groups of saliva samples. Technical bias could be expected, mainly biological bias in the sense of changing saliva viscosity, molecular composition, and obtained volume [30,34]. These issues might be reflected mainly in the evaluation of the results and their comparison. Degradation of salivary protein content occurs relatively shortly after or even during the saliva collection process. Using a protease inhibitor cocktail is recommended for some types of analyses, or the application of sodium azide could help, to some extent, as a stabilizer [35,36]. Stability and the related degradation depends on the nature of the biomarker, one may be more susceptible than another in terms of resistance to degradation [37].

Some methodological differences can be observed regarding the collection and storage of the samples before processing. After collection, saliva samples are usually processed by centrifugation prior to freezing. However, the centrifugation units differ from study to study. Centrifugation serves to remove cell debris and decrease the viscosity of saliva. However, it may affect the results of the analysis, e.g., by lowering the measured marker [38]. Unfortunately, only some studies offered a detailed description of the collection procedure or sample storage. Some studies did not mention the centrifugation process, but only the storage conditions. We also observed a trend in freezing samples before processing, which is why some discrepancies in salivary content may occur.

Before collection, individuals must be asked not to eat and drink beverages such as tea, coffee, or juices. Drinking water is usually allowed but drinking should probably not occur shortly before collection due to the possible dilution of saliva and markers [39]. However, in some studies, an oral water rinse was performed prior to collecting saliva [40]. In some cases, saliva was obtained from the sublingual and parotid regions of the mouth in a non-fasting state, at least 15 min after food or drink consumption, or the participants were instructed to refrain from eating and drinking as well as oral hygiene procedures for at least one hour [41] or even up to three hours prior to sample collection [42]. The interval between last food and drink intake and sampling differs widely between studies.

In general, searching for markers in saliva might be considered research-friendly because of several aspects. The enrolled patients and individuals are experiencing less stress during the collection process, and they may be more willing to participate in research [43]. Establishing general guidelines for the proper conduct for saliva sampling and analysis depending on the analyzed biomarker could enhance saliva research in neurodevelopmental disorders in general.

## 4. Saliva in ASD Research

Searching for biomarkers, especially applicable for the diagnostics of neurodevelopmental disorders, is a truly challenging task. Using blood or amniotic fluid is still considered the gold standard in research and clinical diagnostics. In general, saliva is used for the monitoring of oral and systemic diseases, whereas in the field of other various disorders, saliva is secondary to the other mentioned body fluids or tissues [18,44].

ASD individuals represent a specific group of people, and the saliva collection process in ASD children might be even more difficult than in neurotypical ones. Some ASD individuals may have lower salivary flow, suffer from dry mouth, or have objectively diagnosed xerostomia [45]. However, the collection of saliva is less stressful and exhausting in comparison with blood; thus, it seems more likely that ASD children would cooperate. Whereas unstimulated whole mouth saliva (UWMS) may be difficult to obtain from these individuals in some cases, some studies showed that ASD individuals were capable of providing a higher volume of saliva than controls [46]. Various techniques used for the stimulation of salivation changes the saliva content by changing the concentration of some biomarkers [25,29,34]. Finding the most suitable method of saliva collection may be even more difficult than it seems. The use of, e.g., citric acid for saliva stimulation in one individual may not fit another who would prefer, e.g., stimulation by paraffin chewing, or vice versa. The acceptance of a collection technique also depends on ASD symptomatology but generally does not seem to be problematic [29].

In salivary autism research, a key role could be played by biological variability and/or reproducibility due to several reasons. A tendency toward lower dental care in ASD has been described [47,48]. However, some other studies reported different observations and stated that the means of decayed, missed, filled, and permanent teeth in the primary dentition are lower in children with ASD compared with healthy controls [49]. Surprisingly, a lower incidence of caries was observed in ASD patients compared to healthy controls [46,50]. However, this finding might be caused by more intensive assistance provided by their parents or caregivers that varies from case to case [46,47]. Additionally, it seems that caries incidence in the ASD population might vary depending on the country [51]. Another reason influencing saliva variability might be food interest and related eating behavior such the selectivity, rejections, and aversions presented in ASD [52,53], as well as a strong preference for nutrient-poor foods [54]. Diet affects the overall gut microbiota as well as the oral microbiome [55,56], which has been found to differ in ASD [40]. A bias in the composition of the oral flora caused by preferring some dishes while rejecting others leads to further alternations in salivary biomarkers. Considering these findings, it is difficult to find a homogenous group of ASD patients where the measured differences would reflect variations between the ASD group and the neurotypical group caused by the disorder and not the variations due to external factors. Another common issue, but not exactly a limitation, applicable not solely to saliva research, is the definition of autism and related diagnostic criteria and tools. Although the Diagnostic and Statistical Manual (DSM) is the most commonly used, several studies did not mention the diagnostic process, whether participants were recruited via the pediatrician or neurologist, or if ASD individuals were examined by a psychologist, and for some ASD individuals, no specified diagnostic process was reported [57,58]. However, this is applicable only for a minority of the studies. A relevant, but usually overlooked point, is the ASD diagnostic module. Although low- or high-functioning autism is randomly mentioned, additional information regarding autism status and the module used is missing or its description is rare. This may be another source of the differences between ASD individuals and controls. To find important differences between ASD and the neurotypical population, it is necessary to define ASD very specifically to avoid any source of potential bias.

The following sections summarize and discuss salivary research in relation to ASD and point to the limitations as well as advantages in this field.

### 4.1. DNA Markers

Saliva contains human and oral microbial DNA and RNA usable as biomarkers for the prediction and diagnosis of several diseases, including ASD, but two problematic issues have been described; thus, most of the isolated DNA and RNA originate from the oral microbiome and the amount of isolated human nucleic acids is comparatively low [59]. Therefore, adequate methods for saliva sample collection and processing need to be constructed according to the subsequent analysis. Currently, different specific collection and isolation kits are available to stabilize and extract the human nucleic acids present in saliva samples. A wide range of collection methods and isolation kits have been used in the studies related to ASD biomarker research (Table 1). Using modern and sensitive methods enables the analysis of all types of genetic polymorphisms and expression of genes, and the whole-exome/genome or transcriptome analysis of the patients can be successfully performed in the saliva of patients, including patients with ASD. Considering this, saliva is an adequate source of nucleic acids instead of blood. In addition, a commercial DNA test from saliva samples is now available to everybody.

Large genome-wide association studies focused on the detection of associations between genetic variants and disease produced many non-overlapping genes, which suggest the complex genetic heterogeneity of ASD [60]. Currently, the Simons Foundation Autism Research Initiative (SFARI) Gene Database provides curated information on all known human genes associated with ASD. Many of them are essential during neurodevelopment, play a role as transcriptional factors, or are responsible for chromatin remodeling [61]. Despite the potential of saliva as a source of biological material for genetic and transcriptomic analysis, the nucleic acids isolated from blood have mainly been used in ASD research. To the best of our knowledge, only a small number of studies focusing on salivary nucleic acid analysis have been published.

Braam et al. analyzed six single nucleotide polymorphisms (SNPs) in *CYP1A2* using the saliva samples of ASD patients. They showed that the disappearing efficacy of exogenous melatonin might be caused by a slow melatonin metabolism because of an SNP in the CYP1A2 gene, which may also be associated with the mechanisms that cause autism [58].

With the development of new technologies, whole-exome sequencing and whole-genome sequencing have been widely used in genetic studies in large ASD cohorts [5,62]. The potential of using saliva samples for whole-exome sequencing of ASD patients was confirmed by Feliciano et al. [63]. In this study, exome sequencing and genotyping of 1379 individuals in 457 families with at least one offspring affected with ASD, were performed. They identified variants in genes and loci that are clinically recognized causes or significant contributors to ASD in 10.4% of families without previous genetic findings and the variants that are possibly associated with ASD in an additional 3.4% of families. The analysis indicated 34 genes (listed in Table 1) harboring damaging variants, of which 21 have a known role in ASD or neurodevelopmental disorder. *BRSK2* was suggested as a high-confidence ASD risk gene, which strengthens the association of additional genes (*FEZF2, ITSN1, PAX5, DMWD*, and *CPZ*) in ASD [63]. Their results are also consistent with previous findings supporting the female protective model (explaining the higher prevalence of ASD in males compared to females) [64]. A trend toward a higher frequency of de novo, likely gene-disrupting variants in constringed genes, and a 1.8-fold higher burden of de novo CNVs in ASD cases in females compared with males was observed, even though they were underpowered to detect statistically significant burden differences between the sexes. In contrast, the frequency of rare, inhered CNVs in females and males was similar [63].

For further clarification of the genetic risk factors associated with ASD, the understanding of genetic etiology of ASD, and the possible genetic background of the higher prevalence in males, studies enrolling cohorts of thousands of individuals of both sexes and their families will be needed. Non-invasive saliva sampling, and the possibility of obtaining high-quality genomic data from saliva DNA analysis, may help to increase the engagement and number of participants in future studies.

### 4.2. RNA Markers

Despite genetics playing a key role in ASD etiology, ASD is a heterogenic genetic condition. The genetic architecture of ASD includes common, rare, and de novo variants in at least several hundred genes with interplay among them [65,66]. Therefore, research has shifted and focused more on the investigation of epigenetic factors involved in ASD etiology, or to multi-omics analyses covering many potential predictors of ASD.

MicroRNA (miRNAs) are small non-coding RNA responsible for post-transcriptional negative regulation of gene expression. Some of the identified miRNAs are present in the human brain and they display regulatory functions in different biological processes associated with prenatal and adult neurogenesis, brain maturation, and synaptic plasticity [67,68]. Studies of miRNAs in patients with ASD have demonstrated differential expression patterns and have suggested miRNAs as biomarkers for ASD screening [66,68,69]. However, one of the most common study limitations is the finding that many of the dysregulated miRNAs are not specific for ASD but are common for other neurodevelopmental disorders [68]. The variety of dysregulated miRNAs in ASD individuals is most probably caused by the variety of mechanisms involved in the etiology of ASD. In addition, several additional symptoms are present in ASD that are not considered core symptoms, which affect a remarkable portion of individuals with ASD, including gastrointestinal disturbances or immune disorders.

As we summarized previously, brain tissue, serum, and cultured peripheral lymphoblast for miRNA identification in ASD patients were used in previously published studies [70]. However, according to recent studies, saliva seems to be a convenient biomaterial to find an miRNA–ASD biomarker [71] as well as preferred material from parents of ASD patients [72]. Hicks et al. studied, in-depth, the utility of salivary miRNAs as ASD biomarkers in recent years. In their pilot study, published in 2016, they investigated the potential of salivary miRNAs as diagnostic screening tools for ASD. They identified 14 miRNAs differently expressed in ASD patients compared to neurotypical children using small RNA sequencing (summarized in Table 1) [66]. In the subsequent validation study, they focused on the objective differentiation of children with ASD from their neurotypical peers and children with non-ASD developmental delay using a saliva-based poly-omic RNA panel including human RNAs and microbial RNAs. Thirty-two salivary RNA characteristics (including microbial taxa, mature and precursor miRNAs, piRNAs, and snoRNA, as shown in Table 1) were identified that accurately distinguished ASD status in a training set of 372 children, and displayed 85% accuracy in a separate test set of 84 additional children [71]. In the prospective case-control study, in which it was convenient to minimize the freezing of collected biological material, 14 miRNAs were identified as differentially expressed by comparing children with ASD, peers with typical development, and non-autism developmental delay (Table 1) [73]. They found that a subset of salivary miRNAs is also associated with measures of adaptive and ASD behaviors. In addition to the RNA sequencing approach, a qRT-PCR based analysis of salivary miRNA was published by Sehovic et al. in 2020 [74]. Using this relatively cheap method available in most diagnostic laboratories, fourteen miRNAs were analyzed in typically developing children and children with some type of developmental disorder including ASD. Six miRNAs were found as potential biomarkers. From those, five were differentially expressed within the ASD cohort (Table 1).

In the most recent longitudinal study, the 78 salivary RNAs previously identified in childhood ASD were examined to see if they remain perturbed in older children and/or change with therapeutic intervention [75]. In the older cohort, seven RNAs (four miRNAs, two piRNAs, and one microbial RNA) were associated with scores on The Vineland Adaptive Behavioral Scales 2nd Edition (VABS-II) measuring adaptive behavior, the Autism Spectrum Quotient (AQ) characterizing autistic traits, and the Behavioral Assessment System for Children (BASC) quantifying behavioral strengths in the older group. Within the younger group, twelve salivary RNAs were identified as changing over time in children with ASD receiving intervention (Table 1). In addition, three microRNAs were associated with behavioral scores in the older cohort that changed over time in the younger cohort. These miRNAs are significantly involved in pathways implicated in brain development and function. Conversely, a comparison of the identified miRNAs showed that only a few small RNAs are consistent across the different studies. For example, miR-146b-5p, piR-6463, piR-24085, and miR-148a-5p, associated with behavior parameters and/or change over time in a longitudinal study [75], were also included in a diagnostic panel [71] or identified by RNA sequencing [73]. In addition, miR-23a-3p was similarly identified as overexpressed by RNA sequencing and by qPCR [66,74]. These findings provide pieces of preliminary evidence that RNAs represent promising markers for monitoring developmental trajectory, therapeutic efficiency, and prognosis of ASD. The combination of miRNAs expression profiling and 16S RNA microbiome analysis of saliva from ASD and neurologically unaffected controls identified five differentially expressed miRNAs and a different abundance of specific microbes in ASD compared to controls. Variations in miRNAs and microbes were also associated with behavioral symptoms related to social interaction and communication. From the detected miRNA/bacteria associations, the most relevant was the negative correlation between salivary miR-141-3p expression and Tannerella abundance. miRNAs and microbiome dysregulations found in the saliva of ASD children are potentially associated with the cognitive impairments of patients. These results suggest that miRNA expression and microbiome alteration could be a consequence of ASD symptomatology [76].

The correlation between miRNA expression in ASD and ASD-candidate genes provide evidence of a functional role of miRNA dysregulation in ASD [77]. However, the microbiota can secrete bioactive molecules modifying the host epigenome, and miRNAs are able to selectively regulate the function of the microbiota. Therefore, the approach connecting the analysis of different types of RNA expression and microbiome analysis, together with genetic testing, seems to be the most promising method of obtaining comprehensive results for a better understanding of ASD etiopathology and the potential usage of these molecules for the diagnosis and therapy of ASD and/or alleviation of its clinical symptomatology.

**Table 1:** Studies focused on salivary nucleic acids in autism.

Children No. ASD No. Non-ASD Geographic Region	ASD Dg. Tool/ Module	Age (Years) ASD Non-ASD	Timing	Saliva Collection and Nucleic Acids Extraction	Duration of Sal/Volume	Centrif	Marker	Method	Results ↓↑ in ASD	Ref
ASD: 5 M, 2 F SA: 2 M, 4F Non-ASD (Down syndrome, Opitz GBBB syndrome): 1 M, 1 F NA	NA	12 ± 9.07 6.5 ± 3.64 6 ± 1	noon	Salivette	2 min/0.1–2 mL	1500 g 10 min	DNA- SNPs CYP1A2*1C CYP1A2*1K CYP1A2*3 CYP1A2*4 CYP1A2*6 CYP1A2*1F	qPCR	SNP found in eight of fifteen patients, Allele *1C in two patients Allele *1F in six patients	[58]
457 families with ASD, including 418 simplex and 39 multiplex families NA	ADOS, ADIR/ 1–4	NA	NA	OGD-500 kit (DNA Genotek), DNA extracted in a CLIA-certified laboratory	NA	NA	DNA-exome	WES	CHD8, SCN2A, ADNP, KDM5B, SYNCAP1, ARID1B, SHANK3, DNMT3A, POGZ, FOXP1, CHD2, GIGYF1, ASXL3, BRSK2, KDM6B, CLCN4, ITSN1, MED13L, IRF2BPL, DMWD, QRICH1, MBD5, CPZ, SLC6A8, FEZF2, PAX5, RERE, RNF25, RALGAPB, NR4A2, EGR3, KDM1B, SH3RF3, DPP6	[63]
ASD (comorbid diagnoses included): 19 M, 5 F Non-ASD: 16 M, 5 F NA	DSM-5, ADOS, CARS, Krug Asperger Index/ NA	9.1 ± 2.4 9.2 ± 2.5	Non-fasting state, 10 a.m.–3 p.m.	Rinsing with water, Oragene RNA collection kit (DNA Genotek) Trizol method, purification by RNeasy mini column(Qiagen)	3 mL	NA	RNA-miRNA	Small RNA sequencing	↑ miR-628-5p, miR-335-3p ↓ miR-30e-5p, miR-27a-3p, miR-23a-3p	[66]
Training set: ASD 156 M, 32 F Non-ASD (TD or DD): 122 M, 62 F Validation set: ASD: 45 M, 5 F Non-ASD(TD or DD): 26 M, 8 F NA	DSM-5 criteria/NA	4.5 ± 1.25 4.08 ± 1.33 4.42 ± 1.25 3.83 ± 1.33	NA	Rinsing with water, Oracollect RNA swab (DNA Genotek)/pooled saliva by highly absorbent swab, Trizol method	NA/5–10 sec	Whole saliva	RNA– human, microbial	Small RNA sequencing	Diagnostic panel: miR-92a-3p, mir-146b, miR-146b-5p, miR-378a-3p, miR-361-5p, miR-125-5p, miR-106a-5p, miR-3916, mir-146a, mir-10a, mir-410, piR-24684, piR-9491, piR-27400, piR-6463, piR-29114, piR-12423, piR-24085, piR-15023, SNORD118, Leadbetterarella byssophia, Alphaproteobacteria, Fusarium, Staphylococcus, Clostridiales, Pasteurella multocida, Corynebacterium uterequi, Lactobacillus fermentum, Oenococcus oeni, Streptococcus gallotycus, Ottowia, Yarrowia lipolytica	[71]
Training set: ASD: 161 M, 26 F Non-ASD TD: 76 M, 49 F Non-ASD DD: 48 M, 21 FTest set: ASD: 29 M, 8 F Non-ASD TD: 5 M, 3 F Non-ASD DD: 15 M,10 F NA	DSM-5, ADOS, ADIR, CARS/ NA	4.5 ± 1.25 3.9 ± 1.5 4.2 ± 1.08 3.9 ± 1.67 4.7 ± 1.67 3.7 ± 1.67	Non-fasting state	P-157 Nucleic Acid Stabilizing Swab (DNA Genotek), Trizol method, purification by RNeasy mini column(Qiagen)	5–10 s	NA	RNA- miRNA	Small RNA sequencing	↓miR-28–3p, miR-148a-5p, miR-151a-3p, miR-125b-2–3p, miR-7706 ↑miR-665, miR-4705, miR-620, miR-1277–5p	[73]
ASD: 25 M, 14 F Developmental disorder: 14 M, 2 F Non-ASD: 11 M, 14 F Bosnia and Herzegovina	CARS II/NA	5.07 ± 1.26 5.03 ± 0.98 5.76 ± 1.67	NA	mirVANA isolation kit (Invitrogen™)	NA	NA	RNA- 14 miRNAs	qPCR	↑miR-7-5p, miR-2467-5p ↓miR-23a-3p, miR-32-5p, miR-140-3p, miR-628-5p	[74]
Adolescent cohort: ASD: 37 M, 11 F Non-ASD: 26 M, 22 F Younger cohort: ASD: 20 M, 2 F DD: 6 M, 3 F NA	DSM-5 criteria, ADOS-2/ NA	11.5 ± 4.45 10.4 ± 3.03 4.36 ± 1.08 3.88 ± 1.05	Non-fasting state	Oragene RNA collection kit (DNA Genotek)/Oracollect RNA swab (DNA Genotek), Qiagen miRNeasy MicroKit (Qiagen)	NA	NA	RNA- miRNA	Small RNA sequencing	Associations between behavior and RNA levels: hsa-miR-146b-5p, hsa-miR-29c-3p, hsa-miR-374a-5p, hsa-miR-182-5p, piR-hsa-24085, piR-has-6463, Staphylococcus Changed over time in younger cohort: hsa-let-7e-5p, hsa-miR-125a-5p, hsa-miR-125b-5p, hsa-miR-146b-5p, hsa-miR-148a-5p, hsa-miR-182-5p, hsa-miR-221-3p, Staphylococcus	[75]
ASD: 60 M, 16 F nonASD: 28 M, 11 F NA	ADOS, ADIR/ NA	6.9 ± 1.5 6.9 ± 1.8	No eating or drinking for at least 3 h prior to saliva collection between 8:30 and 10:30 a.m.	Non-stimulated, Qiagen miRNeasy Mini Kit (Qiagen), PureLink Genomic DNA Kit (Thermo Fisher Scientific)	800 µL–4 mL	10,000 rpm, 15 min at 4 °C		NanoString technology, qPCR, 16S RNA sequencing	↑miR-29a-3p and miR-141-3p ↓miR-16-5p, let-7b-5p, miR-451a ↑Rothia, Filifactor, Actinobacillus, Weeksellaceae, Ralstonia, Pasteurellaceae, Aggregatibacter ↓Tannerella, Moryella, TM7-3	[76]

ADOS—Autism Diagnostic Observation Schedule; ADIR—Autism Diagnostic Interview-Revised; ASD—autism spectrum disorder; CARS—Childhood Autism Rating Scale; centrif—centrifugation CNV—copy number variation; CGH—comparative genome hybridization; DD—with developmental delay; Dg tool—diagnostic tool; DSM —Diagnostics and Statistical Manual; dur of sal—duration of salivation; F—female; M—male; miRNA—microRNA; NA—not applicable or mentioned in the text; ref—reference; SA—syndromic autism; SNP—single nucleotide polymorphism; TD—individuals with typical development; WES—whole-exome sequencing., **↓/↑**—downregulated/upregulated in autism; NA—not applicable or mentioned in the text.

### 4.3. Salivary Microbiome in ASD

In addition to the typical ASD-related behavior described above, some pathological traits are common for a high percentage of ASD patients. Gastrointestinal abnormalities are common among children with autism [78,79,80,81,82] and may be listed under ASD-related comorbidities affecting approximately 70% of ASD patients [83]. Studies dealing with the gut–brain axis aimed at finding differences in the microbiotic composition of the fecal flora following Boltes’ hypothesis [55] that abnormal gut microbiota may be involved in the etiology of ASD. In clinical practice, evidence of the microbiota composition influencing gut–brain axis interactions has been obtained from the association of dysbiosis with disorders of the central nervous system. Although the gut–brain axis is slowly gaining in importance and may represent a basis for the research of a wide variety of mental and neurological disorders, it does not yet have clinical application as a diagnostic tool. Investigating the composition of the oral microbiome and its relation to the bacterial overgrowth of the gastrointestinal tract may offer far more options for new potential diagnostic approaches. In comparison to the gastrointestinal system, the oral cavity contains distinct habitats with both hard and soft tissues. The dental plaque contains diverse microorganisms, which seem to be altered in children with ASD [84,85]. Altered numbers of certain microorganisms in the oral cavity of ASD patients may influence ASD-related symptoms and behaviors [86] by provoking the immune system to produce pro-inflammatory molecules that reduce the cohesiveness of the hematoencephalic barrier. Micro-organisms freely entering the central nervous system may influence behavior. The mechanism of this oral microbiome–brain axis or even oral microbiome–gut–brain axis still needs to be explored. One of the potential mechanisms for the entrance of oral micro-organisms to the bloodstream is simply by injury to the oral mucosa, or even mechanical tissue disruption induced by dental care: brushing and flossing. Another potential mechanism may involve the direct transfer of micro-organisms from the oral cavity via the gastrointestinal tract to the gut, inducing the dysbiosis of the gut microbiome [87]. During the past decade, several studies published convincing results proving an altered microbiome in patients with ASD, some of them finding correlates between the oral and gut microbiomes.

Regarding the composition of the oral microbiome of ASD patients, Rothia species were found to be statistically more prevalent in children with ASD. Megasphaera, Moraxella, Neisseria, and Gemella species were, on the contrary, found at significantly lower levels [88]. In general, in the saliva samples of children with ASD, a lower bacterial diversity is observed than in their healthy counterparts. This is consistent, not only with the findings from the gut, but is even more pronounced in dental plaque samples [42]. According to Qiao [42], the genera Haemophilus in saliva and Streptococcus in dental plaque are significantly more abundant in ASD, whereas Prevotella, Selenomonas, Actinomyces, Porphyromonas, and Fusobacterium are reduced. Kong et al. [41] identified an unspecified oral Bacilli genus, the relative abundance of which was significantly different between the ASD and control groups. All the studies confirmed that the oral microbiome composition analyzed from saliva (or even plaque) collected from autistic patients distinguished them from typically developing counterparts [40,41,42,88]. Hicks et al. [40] proved that three of the microbial ratios distinguish ASD children with GI disturbances from those without, as well as ASD children from developmentally delayed children in which ASD was one of the possible diagnoses before undergoing a full diagnostic assessment. Another important factor influencing the oral microbiome is represented by abdominal pain and GI comorbidities including allergies, as well as abnormal dietary habits. Authors also suggested that the oral microbiome might be associated with upper GI health and contribute to abdominal pain. Several oral genera were differentially enriched based on abdominal pain status, including Porphyromonas, Megasphaera, and Haemophilus [41].

Recent studies [89,90] confirmed that the oral and gut microbiome compositions are different in autistic patients, but they yielded inconsistent results in identifying a bacterial genus that might play a major role in autism diagnostics. Only a few studies have analyzed the biomarkers in saliva; moreover, they used limited sample sizes, so verification of the findings described above with larger cohorts are required.

Taken together, when interpreting the results, factors such as missing or decayed and filled teeth and inflammation such gingivitis should be considered. Additionally, the aversion of ASD patients to dental procedures and hygiene might be one mechanism of oral dysbiosis [42]. Even though Qiao [42] did not confirm a correlation between phylotypes and decayed, missing, or filled teeth, a positive association of the presence of *Aggregatibacter segnis* with bleeding on probing, gingival index, and periodontitis.

Other factors relevant to the oral microbiome are listed in the paper by Hicks [40]: (a) demographic information (age, sex, ethnicity, and body mass index), (b) oral/GI factors (time of collection, time of last meal, time of last tooth brushing, probiotic use, history of GI disturbance, medical/food allergies, and dietary restrictions), and (c) medical history (birth age, birth delivery route, birth weight, asthma status, and vaccination status).

### 4.4. Hormones

Steroid hormones in saliva represent one of the first markers routinely detected in saliva in general and they are one of the most analyzed markers related to ASD. This is probably due to the bias toward a higher number of diagnosed males compared to female patients.

The most used detection technique so far is RIA or ELISA, offering detection of only a limited number of hormones, focused on major intermediates and products. The sensitivity and specificity of, e.g., ELISA for the analysis of saliva need improvement [91] with regard to the targeted biomarker and the type of ELISA [92]. Accurate and more sophisticated techniques enabling the detection of a broader spectrum of markers, or especially hormones, in saliva are needed. Currently, chromatography-mass spectrometry enables the analysis of a cascade of hormones and provides almost a complete picture of the hormonal profile. Unfortunately, as the nature of some molecules in terms of their structure and size, e.g., aldosterone, does not allow their detection using these methods, methods such as RIA are helpful and are used in these cases [93].

Since ASD represents a multifactorial disorder with a strong genetic background, the analysis of only a few hormones may not be helpful in revealing the pathophysiology or looking for diagnostic markers. Although the available studies are difficult to compare due to the different ASD groups studied, they have some specific features in common, e.g., measurement of cortisol (Table 2) [94]. Differences between the ASD and control groups, e.g., in cortisol concentrations, were not always observed in various studies performed in pre-pubertal children or pubertal individuals [93,95]. Although morning cortisol did not differ between groups [96], evening cortisol was found to be higher in ASD children compared to controls [97] and several studies pointed to a higher variability in the cortisol circadian rhythm. This condition might be related to behavioral adaptation, stress, symptoms, or low-/high-functioning autism, and higher circadian salivary cortisol variability was observed in ASD children [96,98,99]. Additionally, the salivary collection process may affect these results.

Other commonly measured hormones are male androgens, which have been found to play a role in autistic trait development [100,101]. Their higher concentrations were found in the amniotic fluid of women having an ASD child or women with pathological conditions such as polycystic ovary syndrome, associated disrupted hormonal status, and an excess of androgens [3,16]. However, the measurements using saliva have not unequivocally demonstrated differences between autistic and neurotypical individuals in the sense of salivary hormones. Higher levels of salivary dehydroepiandrosterone (DHEA) and androstenediol were observed in early pre-pubertal boys. Interestingly, these hormones were higher in pre-pubertal ASD girls. DHEA was also higher in female ASD children compared to controls [93]. Salivary testosterone (TST) in adult and pubertal males was not found to be different in ASD vs. controls [95]. Furthermore, the salivary baseline levels of TST and estradiol were comparable and did not differ between adult ASD women and matched controls [102]. Unfortunately, only a few studies were devoted to the assessment of a wider cascade of hormones in saliva. However, this is not just limited to saliva as a potential diagnostic fluid, but also to blood. A detailed detection of steroids such as the one performed by Majewska et al. [93] offered a complex view of the differences allowing us to obtain the whole hormonal profile, and thus, reveal more about the pathophysiology of ASD.

Analyses of salivary oxytocin do not provide obvious results because, whereas one study observed lower level of this hormone in ASD [95], another observed a trend toward a higher level of baseline oxytocin in ASD compared to controls [102], and another found no difference between ASD and controls [103].

The limitation regarding the use of standard techniques such as ELISA or RIA is their technical variability. Although the kits may vary from manufacturer to manufacturer, comparable concentrations of markers detected in saliva would be expected. However, these considerations pose problems in addition to the already previously mentioned limitations such as variations in saliva sampling, their processing, a wide age variance in the analyzed groups, and, possibly, the definition of the analyzed group. One of the problems might be the stability of hormones under different collection and storage conditions. Repeated thawing and freezing of the saliva samples affects the stability of several hormones in terms of, e.g., their concentration. Although some may stay unchanged, e.g., progesterone, the concentration of others, e.g., cortisol, may decrease [104]. However, this finding is also not uniform, differing between studies; a stable concentration of cortisol was found [105]. The stability is determined by the chemical property of every marker and although cortisol was observed to be stable under long-term conditions, the stability of salivary alpha-amylase was lower. This points to the need for a long-term longitudinal study where newer samples are evaluated together with those stored for a longer time [106], and the same collection procedure has to be maintained throughout the project [107]. Treatment of saliva samples before storage is rare, but using sodium azide protected from degradation of hormones by keeping the concentration of hormones stable over time [104].

One factor that should be considered is the age of the participants. Having equal age groups is not the same as having age-matched individuals either in the analyzed or the control group. Some markers, especially hormonal ones, are closely related to age, thus, this fact must be critically considered. Likewise, the effect of sex in hormonal studies is usually overlooked. Even when studies analyzed both males and females, they usually did not look at the differences between them and analyzed them separately [93], or these differences were described in other types of samples, which were analyzed in addition to saliva samples [108]. In some cases, only a low number of females were included, so both males and females were grouped either in the ASD or control group [96,109,110,111]. However, these limitations may not be related solely to saliva but also to blood analysis. The hormonal research performed on saliva samples is summarized in Table 2.

**Table 2:** Hormones in saliva of autistic individuals.

Children No. ASD No. CTRL Geographic Region	Dg. Tool/ ASD Module	Age (Years) ASD CTRL	Timing	Saliva	Duration of Sal/Volume	Centrif	Marker	Concentration ASD, CTRL	Method	Results ↓↑	Ref
High functioning ASD 37 M 2 F NA	ADIR/ NA	8.64 ± 1.50	NA	UWMS Salivette Sorbette	NA 45 s	NA	µg/dL Cortisol	RM ANOVA F(2,37) = 0.53 Mean (SD) 0.089 (0.045) 0.084 (0.036) 0.084 (0.035)	ELISA	*p* = 0.592	[29]
ASD M 23 CTRL M 21 ASD F 22 CTRL F 16 22 markers were analyzed, also in saliva of females Poland	DSM-IV CARS/ NA	3.7 ± 0.1 3.5 ± 0.1 3.9 ± 0.2 3.4 ± 0.1 8.2 ± 0.2 8.4 ± 0.2 7.7 ± 0.2 8.4 ± 1.4	8–10 a.m.	Salivette	0.15–5 mL	Frozen 3000- 4000 g, 10 min	nmol/L DHEA-C Androsterone-C Pregnenolone Allopregnanolone DHEA DHEA-C Androstenediol Etiocholanolone Epiandrosterone	Mean (±SEM), ASD, CTRL boys 69.66 (30.05); 7.702 (1.440) 6.972 (1.767); 3.0.57 (0.770) ASD, CTRL males 4.454 (0.682); 1.295 (0.133) 0.177 (0.029); 0.030 (0.008) 5.522 (1.934); 0.880 (0.106) 560.05 (278.11); 39.06 (23.82) 1.746 (0.206); 0.637 (0.091) 0.0702 (0.014); 0.015 (0.003) 0.402 (0.223); 0.075 (0.011)	GC-MS RIA	↑ ASD (0 < 0.05) ↑ ASD (*p* = 0.010) ↑ ASD (*p* < 0.05) ↑ ASD (*p* < 0.05) ↑ ASD (*p* < 0.01) ↑ ASD (*p* < 0.01) ↑ ASD (*p* < 0.01) ↑ ASD (*p* < 0.01) ↑ ASD (*p* < 0.01)	[93]
ASD 20 M ASDanx 32 M CTRL 23 M United Kingdom	ADOS-G ADI-R SCQ/ NA	13.9 (1.9) 12.9 (2.0) 12.8 (2.0)	1:20 p.m. 1:40 p.m.	Salivette	NA	NA	Unit NA Cortisol	Log-transformed, F(2,72) = 0.06 F(2,72) = 0.11 Mean (SD) 13:20 p.m., 13:40 p.m. 1.77 (0.53); 1.82 (0.50); 1.80 (0.46) 1.56 (0.41); 1.54 (0.40); 1.59 (0.34)	Immulite	-	[94]
ASD 49 M CTRL 28 M The Netherlands	DSM-V DISC-IV/ NA	15.0 ± 1.8 15.9 ± 2.1	6:45 a.m.–12:30 p.m.	UWMS	NA/6 mL	Non-C	Oxytocin Testosterone Cortisol	Calculated as Z scores (SD) −0.22 (0.89); 0.49 (0.97) −0.26 (0.99); −0.04 (0.74) −0.19 (1.02); 0.02 (0.75)	RIA	Oxytocin ↓ ASD (*p* < 0.01)	[95]
ASD 30 M, 6 F CTRL 23 M, 4 F NA	ADOS DSM-IV/ NA	10.2 ± 1.96 9.71 ± 1.54	M1—waking M2—30 min after A—approx. 3 p.m. E-30 min bedtime	UWMS	NA	Frozen 2558 g 15 min	ng/mL Cortisol	Diagnosis-F(1,128) = 4.78 M1-F(1,103) < 1 M2-F(1,123) < 1 A-F(1,110) < 1 E-F(1,122) = 7.86 Mean (SD) M1—2.86 (1.20); 2.54 (1.11) M2—3.58 (1.60); 3.23 (1.46) A—1.40 (1.73); 0.83 (0.35) E—0.47 (0.59); 0.18 (0.14)	Coat-A-count RIA	*p* = 0.031 *p* = 0.50 *p* = 0.76 *p* = 0.40 ASD *p* = 0.006	[96]
ASD 57 M, 7 F CTRL 42 M, 7 F NA	ADOS DSM-V/ NA	12.02 11.17	M1—waking M2—30 min after AFT—1–4 p.m. EVE-30 min bedtime	straw	1 mL	Frozen 3640 rpm 15 min	ng/mL Cortisol-C	dg x C—F(1,103) = 1.73, η^2^ = 0.02 dg x time F(2.20,226.99) = 4.08, η^2^ = 0.04 EVE—F(1,103) = 6.88, η^2^ = 0.06 Log-transformed Mean (SD) M1—0.33 (0.25); 0.34 (0.26) M2—0.44 (0.31); 0.48 (0.19) AFT—−0.05 (0.32); −0.10 (0.28) EVE—−0.60 (0.40); −0.81 (0.41)	Coat-A-count RIA	*p* = 0.19 *p* = 0.02 ↑ ASD (*p* = 0.01) *p* = 0.82 *p* = 0.45 *p* = 0.42 ↓ ASD *p* = 0.01	[97]
LF ASD 36 M,19 F CTRL 22 M, 12 F France	DSM-5 ICD-10 CFTMEA ADOS-G ADI-R/ module 1	11.3 ± 4.1 11.7 ± 4.9	8 a.m. 11 a.m. 4 p.m. 0 a.m. 8 a.m. next day	swab	>1 min	NA	µg/dL Cortisol	Mean (SEM) 0.803 (±0.134); 0.620 (±0.070) 0.529 (±0.94); 0.429 (±0.089) 0.365 (±0.048); 0.201 (±0.018) 0.305 (±0.077); 0.104 (±0.002) 1.209 (±0.167); 0.620 (±0.071) RM measures of variance Group–F(1,31) = 20.74 Time–F(4,124) = 54.07 Group by time –F(4,124) = 1.59	ELISA	*p* = 0.2318 *p* = 0.2154 ↑ ASD *p* = 0.0017 ↑ ASD *p* = 0.0001 ↑ ASD *p* = 0.0016 *p* < 0.001 *p* < 0.001 *p* = 0.187	[98]
ASD 12 M CTRL 10 M California	ADOS DSM-IV/ NA	8.5 9.2	1–3 p.m. (1) Eve—30 min of bedtime M-30 min post-waking 1–3 p.m. (2) Eve (2) M (3)	Chewing gum, straw	30 s	Frozen 6000 rpm 10 min	nmol/L Cortisol	Log-transformed NA	RIA	Overall cortisol level—*p* = 0.45 Daily variations *p* > 0.08 (for interactions between ASD and both time of day contrasts)	[99]
ASD 16 F CTRL 29 F NA	DSM-V ICD-10/ NA	18–50 29.9 ± 8.4 28.7 ± 9.0	NA	UWMS	NA	NA	pg/mL Estradiol Testosterone Oxytocin	Mean (SD) 1.0 (±0.3); 1.2 (±0.5) 70.3 (±24.9); 69.4 (±21.4) 3.1 (±0.5); 2.8 (±0.16)	ELISA RIA	*p* = 0.19 *p* = 0.91 *p* = 0.064	[102]
ASD 17 M CTRL 24 M Japan	DSM-V DISCO/ NA	27.4 ± 7.2 29.0 ± 9.8	NA	Salivette	NA	NA	pg/mL Oxytocin	36.2 (13.2); 43.6 (17.0)	ELISA	*p* = 0.154	[103]
ASD 11 M, 9 F CTRL 15 M,13 F NA	ADOS DSM-IV-TR CARS/ NA	3–10	Before blood draw 20 min after 40 min after	Salivette Sorbette	NA	NA	µg/dL; log-transformed Cortisol	Mean (SEM), ASD; CTRL 0.242 (0.061); 0.175 (0.024) 0.426 (0.107); 0.132 (0.018) 0.329 (0.111); 0.011 (0.018)	EIA	*p* = 0.242 *p* = 0.014 ↑ ASD *p* = 0.057	[104]
ASD M 22, F 4 CTRL M 23, F 3 NA	ADOS-G ADI-R DSM-IV/ NA	Age in months 45.1 ± 8.9 39.4 ± 10.5	Waking within 30 min Midday 2 p.m. Bedtime within 30 min Waking 2 Midday 2 Bedtime 2	Kool-Aid drink swab	0.5–1.0 mL		nmol/L Cortisol	Coefficient 0.17 (CI, −0.08–0.49)	ELISA Kinetic reaction assay	*p* > 0.23	[109]
ASD 21 M, 1 F CTRL 19 M, 3 F California	ADOS ADI DSM-IV/ NA	8.81 ± 1.90 9.35 ± 1.75	M1—waking A1—afternoon (1–4 p.m.) E1—30min bedtime Collection 6 days during 2 weeks	chewing gum	NA	Refrigerated until the end of collection Frozen 6000 rpm 10 min	nmol/L Cortisol M1 A1 E1 M2 A2 E2 M3 A3 E3 M4 A4 E4 M5 A5 E5 M6 A6 E6	Mean (SD), ASD; CTRL 15.60 (16.90); 11.40 (6.87) 3.90 (2.92); 3.14 (1.34) 2.31 (3.39); 1.46 (0.72) 10.30 (3.39); 9.83 (4.63) 3.25 (2.17); 3.03 (1.57) 2.53 (3.18); 1.26 (0.21) 11.70 (7.07); 12.10 (5.97) 3.60 (3.20); 3.22 (1.64) 4.94 (12.0); 1.40 (0.30) 11.20 (5.02); 11.30 (5.81) 4.84 (4.94); 2.97 (1.30) 2.50 (2.86); 1.31 (0.46) 8.88 (4.14); 11.60 (6.50) 4.76 (3.68); 3.40 (1.54) 2.05 (1.63); 1.53 (0.57) 8.15 (6.12); 10.70 (4.29) 4.60 (7.72); 3.17 1.77) 3.07 (4.56); 1.53 (0.57)	RIA	E concentration over 6 days *p* = 0.021 E2-E5 ↑ ASD *p* < 0.04 Multiple regression Time *p* > 0.05 Sleep *p* > 0.05 Age *p* > 0.05	[110]
ASD 50 CTRL 50 NA	NA/ NA	6–12	2× 8–8:30 a.m. 2× 4–4:15 p.m.	UWMS	2 min drooling intervals/2mL	Non-C	Cortisol (NA) 1st day morning 1st day evening 2nd day morning 2nd day evening	Mean (SD) 57.12 (14.108); 96.16 (15.694) 101.4 (17.647); 66.39 (11.735) 57.96 (13.883); 96.73 (16.569) 102 (17.827); 66.55 (12.099)	ECL	↓ ASD (<0.001) ↑ ASD (<0.001) ↓ ASD (<0.001) ↑ ASD (<0.001)	[111]

ADOS-G—Autism Diagnostic Observation Schedule-Generic; ADIR—Autism Diagnostic Interview-Revised; ASD—autism spectrum disorder; ASDanx—ASD with an anxiety disorder; AT a/b—5-androstene-3b;7a/b;17b-triol; CTRL—control; CARS—Childhood Autism Rating Scale; Dg—diagnosis; Dg tool—diagnostic tool; DISC-N—Diagnostic Interview Schedule for Children; DISCO—Diagnostic Interview for Social and Communication Disorders; DHEA—dehydroepiandrosterone; DHEA-C—dehydroepiandrosterone conjugate; DSM—diagnostics and statistical manual; ECL-electrochemiluminescence; GxT—group by time interaction; IQR—inter-quartile range 25–75%; LC-MS/MS—liquid chromatography-mass spectrometry; LF ASD—low functioning autism; NA—not applicable or mentioned in the text; Non-C—non-centrifuged; RM ANOVA—repeated measures ANOVA; RIA—radioimmunoassay; SCQ—Social Communication Questionnaire; SD—standard deviation; SEM—standard error mean; UWMS—unstimulated whole mouth saliva; ↑/↓—higher/lower concentration in autism; NA—not applicable or mentioned in the text.

### 4.5. Protein and Inflammatory Markers

Other factors possibly contributing to the development of ASD is inflammation and a disrupted function of the organism’s immune system. The mother’s immune response to a pathogen is responsible for changes in fetal brain development [112]. A causal relationship between uterine immunity and an increased risk of neurodevelopmental disorders, such as ASD, is supported by multiplying clinical, epidemiological, and experimental findings [113]. Recent studies on mice and humans showed the role of uterine immunity during pregnancy in the determination of the health trajectory of the offspring and the significant impact on cognitive function and mental health. The main contributor to heterogeneous pathological and behavioral phenotypes associated with ASD is IL-17a-producing Th17 T cells. Other cytokines, including IL-6 and TNFα, were implicated as critical effectors of uterine immune activation and ASD severity [114].

The profiles of pro-inflammatory cytokines are also altered after birth in individuals with ASD compared to healthy control subjects. The published studies frequently used convenient biomaterials, whole blood, plasma, and serum. A meta-analysis of 38 published studies provided evidence for higher concentrations of IFN-γ, IL-1β, IL-6, and TNF-α in autistic patients and pointed to the interaction of latitude, age, and sex with peripheral alterations in the associated pro-inflammatory cytokines [115].

Although saliva as a source of biological material has many advantages including non-invasiveness and cost-effectiveness, its use in protein and inflammatory markers of ASD research is limited (Table 3). The relative low number of studies could be caused by issues related to the collection and processing of saliva samples explained in previous sections. Even though the saliva proteome, which refers to all proteins found in the mouth, may overlap with the blood and cerebrospinal fluid (CSF) proteome (approximately 2290 saliva proteins compared to 2698 blood proteins), protein levels may be more difficult to detect in saliva than in blood and CSF because of their dilution and variable concentration [44,116]. The high-abundance proteins such as albumin and amylase represent at least 60% of the human salivary proteins. Thus, the detection of low-abundance proteins represents a significant analytical challenge for saliva proteome characterization and discrimination between physiological and pathological conditions. Nevertheless, no standard proteomic protocol for analysis of the entire saliva content is not currently available. The salivary proteome was initially investigated in several diseases by NMR spectroscopy, as well as gas and liquid chromatography-mass spectrometry [117].

One of the first proteomic studies published in 2008 focused only on the detection of phosphorylation of salivary proteins and not on the measurement of differences in their levels in ASD. Its results showed that the proteins histatin, statherin, and proline-rich phosphopeptide were hypo-phosphorylated in the saliva of ASD children relative to control subjects. The authors further noted that a subset of children with ASD had the hypo-phosphorylated phenotype and a tendency to be of normal-to-borderline intellectual development [118].

With the aim of identifying initial biomarkers in children with ASD, the optimization of mass-spectrometry-based salivary proteomic analysis was performed by Ngounou Wetie et al. [119]. Their results showed that nano liquid chromatography-tandem mass spectrometry (nano LC-MS/MS) of the salivary proteome could help expose the biomarkers for ASD; statistically significant differences were detected in several salivary proteins, e.g., the elevation of prolactin-inducible protein, lactotransferrin, Ig kappa chain C region, Ig gamma-1 chain C region, neutrophil elastase, polymeric immunoglobulin receptor, and deletion in malignant brain tumors 1, between ASD and normal, healthy individuals. In addition, all these factors could be elevated as a part of the immunological reaction and increase in response to inflammation, which supports the role of inflammation in ASD etiology.

In the next study published by the same group of authors, a two-dimensional polyacrylamide gel electrophoresis paired with nano LC-MS/MS was used for the determination of the relative abundance of saliva proteins and investigation of the differences between the salivary proteomes of children with ASD and matched controls. They detected significant differences in many proteins and highlighted the biological relevance of these proteins in ASD. Many of the differentially expressed proteins were previously linked to ASD or suggested as risk factors of ASD at the genetic level (e.g., alpha-amylase, CREB-binding protein, p532 protein, and proto-oncogene FRAT1). Some others are involved in the pathological pathways implicated in ASD causality such as oxidative stress, lipid and cholesterol metabolism, immune system disturbances, and inflammation [120].

For a better understanding of the relationships between specific cytokines and the risk of ASD, the salivary levels of IL-1β, IL-6, IL-8, regulated on activation, normal t-cell expressed and secreted (RANTES), eotaxin, monocyte chemoattractant protein-1 (MCP-1), and TNFα were analyzed on a Luminex with custom-designed seven-plex kits in a study published in 2020 [121]. The results showed a significantly lower level of RANTES in ASD children compared to children with typical development. The salivary levels of IL-1, MCP-1, and TNFα correlated positively with age in the control group but not in the ASD group. Significant differences were also detected between the RANTES salivary level and aggression and gait disturbances, between IL-8 salivary level and fixations and stimulations, and between salivary IL-1β and no active speech.

Published data proved the utility of saliva for proteomic analysis, including inflammatory markers, and its potential to advance the understanding of ASD etiology as well as provide novel treatment for ASD. However, studies with a larger set of samples with well-characterized subjects and conditions of saliva sampling and processing (including latitude, sex, age, time of collection, and diet) [122,123] need to be performed to confirm these findings and implement them into clinical practice.

**Table 3:** Protein and inflammatory markers in autism.

Children No. ASD No. CTRL Geographic Region	ASD Dg. Tool/ Module	Age (Years) ASD CTRL	Timing	Saliva	Duration of Sal/ Volume	Centrif	Marker	Method	Results ↓↑ in ASD	Ref
ASD 27 CTRL 23 NA	NA	2–15 years	NA	NA	NA	NA	proteome	NA	Hypophosphorylation of histatin, statherin, proline-rich phosphopeptide	[118]
ASD 6 M, 0 F CTRL 6 M, 0 F NA	DSM-5/ NA	11.67 ± 2.49 9.5 ± 2.22	NA	Passive drool into a straw and collection cup	1–2 mL	20 min	proteome	nanoLC-MS/MS	↑ prolactin-inducible protein, lactotransferrin, Ig kappa chain C region, Ig gamma-1 chain C region, neutrophil elastase, polymeric immunoglobulin receptor, deleted malignant brain tumors 1 ↓ acidic proline rich phosphoprotein, Statherin, histatin-1	[119]
ASD 6 M, 0 F CTRL 6 M, 0 F NA	DSM-5/ NA	11.67 ± 2.49 9.5 ± 2.22	NA	Passive drool into a straw and collection cup	1–2 mL	14000 rpm 10 min	proteome	2D-PAGE, nanoLC-MS/MS	↑ proto-oncogene FRAT1, Ig alpha-1 chain C region, immunoglobulin heavy chain constant region alpha-2 subunit, V-type proton ATPase subunit C 1, Kinesin family member 14, Integrin alpha 6 subunit, growth hormone regulated TBC protein 1, parotid secretory protein, Prolactin-inducible protein precursor, Mucin-16, Ca binding protein MRP14 ↓ alpha-amylase, CREB-binding protein, p532, Transferrin variant, Protein-L-isoaspartate O-methyltransferase domain-containing protein 1 isoform 3, Chain A of Human Pancreatic Alpha-Amylase In Complex With Myricetin, V-type proton ATPase subunit C 1, Ig J-chain, Zn alpha2 glycoprotein, Glutamate-rich protein 6B, Immunoglobulin heavy chain variable region, Albumin protein, Sperm activating protein subunit I-Apo A1-SPAP-subunit I, Zymogen granule protein 16 homologue B precursor, Putative lipocalin 1-like protein 1, cystatin D, plasminogen	[120]
ASD 18 M, 1 F CTRL 15 M, 4 F Poland	DSM-5, ADOS-2/ NA	6.78 ± 2.8 6.84 ± 2.52	9–11 a.m.	Salivette collection tube	5 min/>1 mL	4500 g 10 min	Eotaxin, RANTES, IL-6, IL-8, IL-1β, TNF-α, MCP-1	Luminex	↓ RANTES	[121]

ASD—autism spectrum disorders; CTRL—control; Dg. tool—diagnostic tool; DSM—diagnostics and statistical manual; ADOS—Autism Diagnostic Observation Schedule; F—female; M—male; NA—not applicable or mentioned in the text; nanoLC-MS/MS—nano liquid chromatography-tandem mass spectrometry; RANTES—Regulated on Activation Normal T-cell Expressed and Secreted; MCP-1—monocyte chemoattractant protein-1; 2D-PAGE—two-dimensional polyacrylamide gel electrophoresis; ↑/↓—higher/lower concentration in autism; NA—not applicable or mentioned in the text.

### 4.6. Oxidative Stress Markers

Oxidative stress, described as an imbalance between pro-oxidants and anti-oxidants in favor of pro-oxidants, was found to be associated with ASD [124]. As with any other marker, markers of oxidative stress (OS) and related anti-oxidant status in saliva are not in the spotlight in ASD research (Table 4). Techniques for the assessment of these markers require a higher volume of saliva. Markers of OS in saliva are closely related to oral health status, caries, and oral hygiene. A significantly lower level of total anti-oxidant capacity was observed in UWMS in children with autism in comparison to controls, whereas no differences were observed between individuals with low and high functioning autism [125]. Additionally, a lower level of glutathione was observed in saliva stimulated by Arabic gum in pubertal children with autism [126]. Another study observed a higher level of 15-F2t-Isoprostane, a marker of lipid peroxidation, in the saliva of autistic children compared to control individuals [127]. All these findings point to the protentional oxidative stress presented in the saliva of ASD individuals [128], but still oral hygiene and cleaning habits have to be considered [125]. Markers of OS are in general accompanied by intra-individual variability, so their application in larger study groups is recommended [128]. In addition to OS markers, the level of salivary sialic acid was found to be lower in ASD individuals in comparison with controls [129].

### 4.7. pH, Ions, and Buffering Capacity

The salivary pH is not a routinely detected or measured parameter. The standard salivary pH ranges from 6.8 to 7.2 and varies during the day, being affected by, e.g., consumption, saliva flow rate, or diet [130,131]. However, its changes or differences from usual values might also point to oral health abnormalities. As mentioned above, ASD children have specific eating habits, causing these differences. Depending on the food preferred, differences between ASD individuals and controls would be expected. A higher salivary pH was observed in the stimulated saliva of ASD individuals in comparison with controls. A higher salivary flow rate in ASD patients was also observed [126]. Another study found no differences in pH between the unstimulated saliva of ASD patients and controls [57].

The stimulation of salivation induces changes in pH, which becomes more alkaline [103]. Thus, in this case, a higher flow rate in ASD, even under stimulating conditions, could contribute to this finding. However, salivary pH may be related to caries; a lower pH was found in an ASD group with caries compared to the caries-free one [126]. Conversely, other studies found no differences in the salivary pH between ASD and control groups despite worse oral hygiene observed in the ASD group [125,132]. Related to pH is the buffering capacity of saliva, which increases with salivary stimulation [131]. In relation to ASD research, contradictory results have been published: one study observed a lower buffering capacity in the ASD group compared to controls and the other one found no differences between these groups [48,57]. These data show that the diagnostics of ASD would not benefit from buffering capacity measurement regarding the diagnosis of ASD, but it may be a valuable indicator of oral health in ASD children. Studies regarding this aspect are mentioned in detail in Table 4.

## 5. Conclusions

The advantages of saliva in autism research and its potential application in clinical practice cannot be denied. As mentioned at the end of every section, several factors must be considered and a strategy for salivary research and saliva use has to be established. In general, technical and methodological aspects are important to consider with respect to the analyzed marker. Regarding the field of neurodevelopmental disorders, especially ASD, homogenous groups defining individual modules, strictly focused on age and gastrointestinal and food history, would be recommended to minimize potential variability. The direct application of saliva in the diagnostic process depends on the finding of a possible biomarker of ASD. However, the discovery of this biomarker is not the only goal of salivary research: general research performed on a variety of biological samples is also required. Afterward, saliva may be considered as a non-invasive alternative tool using all its advantages. Considering all these factors, the detection of markers from various points of view would help to further our understanding of ASD and reveal the etiopathogenesis behind these disorders.

## Figures and Tables

**Table 4:** Non-protein markers and parameters in saliva of autistic individuals.

Children No. ASD No. CTRL Geographic Region	ASD Dg. tool/ Module	Age (years) ASD CTRL	Timing	Saliva	Duration of sal/Volume	Centrif	Marker	Concentration Mean (±SD) ASD CTRL	Method	Results ↓↑	Ref
ASD 34 M CTRL 34 M Venezuela	NA/ Grade 1–2	8.12 ± 1.92	NA	NA	NA	NA	nmol/L Ca P	0.621 ± 0.35; 0.89 ± 0.51 6.17 ± 4.22; 5.51 ± 4.86		↓ ASD, *p* = 0.013	[50]
ASD 20 M, 10 F CTRL sib 8 M, 22 F Karnataka	NA/ NA	6–12	9–11 a.m.	UWMS	1 min	NA	mL/min Salivary flow pH	0.8 (0.35); 0.78(0.47) 6.49 (0.58); 7.08(0.62)	pH strip	*p* > 0.05 ↑ ASD *p* < 0.05	[57]
ASD 101 CTRL 50 sib NA	NA/ NA	6–12	9–10 a.m.	UWMS	NA	NA	pH TAC µg/mL	Median 7; 7 Median 5.7; 38	pH paper phospho molybdate method	*p* = 0.376 ↓ ASD *p* < 0.001	[125]
ASD 40 M CTRL 40 M Iraq	NA/ NA	12–15	NA	Arabic gum	10 min	1000 rpm 10 min	GSH pH flow rate Cu Zn	0.89 ± 0.58; 2.20 ± 1.10 7.45 ± 0.48; 6.9 ± 0.57 1.70 ± 0.25; 1.51 ± 0.30 31.58 ± 11.74; 25.21 ± 9.15 73.66 ± 17.67; 64.05 ± 16.64	ELISA pH meter atomic absorption spectrometer	↓ ASD *p* < 0.001 ↑ ASD *p* < 0.001 ↑ ASD *p* = 0.003 ↑ ASD *p* = 0.008 ↑ ASD *p* = 0.014	[126]
ASD 36 M, 10 F 28 no treatment, 18 treated CTRL 21 M, 9 F NA	DSM-V CARS AuBC NA	5.5 ± 2.05 5.35 ± 2.15	8–9 a.m.	Residual WS	NA/1.5–2 mL	3000 g 15 min	Sialic acid (mmol/L)	0.102 (±0.062) 0.100 (±0.099) 0.160 (±0.097)	Sialic acid assay kit	↓ ASD *p* = 0.027	[129]

ASD-autism spectrum disorder, CARS—Childhood Autism Rating Scale, CTRL—control, Dg. tool—diagnostic tool, DSM—diagnostics and statistical manual, F—female, GSH—reduced glutathione, M—male, NA—not applicable or mentioned in the text, NS—non-stimulated saliva, S—stimulated saliva, sib—siblings, TAC—total antioxidant capacity, UWMS—unstimulated whole mouth saliva, WS—whole saliva, ↑/↓—higher/lower concentration in autism.

## Data Availability

Not applicable.

## References

[1] Hodges H., Fealko C., Soares N. (2020). Autism spectrum disorder: Definition, epidemiology, causes, and clinical evaluation. Transl. Pediatrics.

[2] Maenner M.J., Shaw K.A., Baio J., Washington A., Patrick M., DiRienzo M., Christensen D.L., Wiggins L.D., Pettygrove S., Andrews J.G. (2020). Prevalence of Autism Spectrum Disorder among Children Aged 8 Years—Autism and Developmental Disabilities Monitoring Network, 11 Sites, United States, 2016. MMWR Surveill. Summ..

[3] Kosidou K., Dalman C., Widman L., Arver S., Lee B.K., Magnusson C., Gardner R.M. (2016). Maternal polycystic ovary syndrome and the risk of autism spectrum disorders in the offspring: A population-based nationwide study in Sweden. Mol. Psychiatry.

[4] Imbriani G., Panico A., Grassi T., Idolo A., Serio F., Bagordo F., De Filippis G., De Giorgi D., Antonucci G., Piscitelli P. (2021). Early-Life Exposure to Environmental Air Pollution and Autism Spectrum Disorder: A Review of Available Evidence. Int. J. Environ. Res. Public Health.

[5] Iossifov I., O’Roak B.J., Sanders S.J., Ronemus M., Krumm N., Levy D., Stessman H.A., Witherspoon K.T., Vives L., Patterson K.E. (2014). The contribution of de novo coding mutations to autism spectrum disorder. Nature.

[6] Jansakova K., Hill M., Celarova D., Celusakova H., Repiska G., Bicikova M., Macova L., Ostatnikova D. (2020). Alteration of the steroidogenesis in boys with autism spectrum disorders. Transl. Psychiatry.

[7] Fombonne E. (2009). Epidemiology of pervasive developmental disorders. Pediatr. Res..

[8] Elsabbagh M., Divan G., Koh Y.J., Kim Y.S., Kauchali S., Marcin C., Montiel-Nava C., Patel V., Paula C.S., Wang C. (2012). Global prevalence of autism and other pervasive developmental disorders. Autism Res. Off. J. Int. Soc. Autism Res..

[9] Hansen S.N., Schendel D.E., Parner E.T. (2015). Explaining the increase in the prevalence of autism spectrum disorders: The proportion attributable to changes in reporting practices. JAMA Pediatr..

[10] Kim Y.S., Leventhal B.L., Koh Y.J., Fombonne E., Laska E., Lim E.C., Cheon K.A., Kim S.J., Kim Y.K., Lee H. (2011). Prevalence of autism spectrum disorders in a total population sample. Am. J. Psychiatry.

[11] Christensen D.L., Maenner M.J., Bilder D., Constantino J.N., Daniels J., Durkin M.S., Fitzgerald R.T., Kurzius-Spencer M., Pettygrove S.D., Robinson C. (2019). Prevalence and Characteristics of Autism Spectrum Disorder among Children Aged 4 Years—Early Autism and Developmental Disabilities Monitoring Network, Seven Sites, United States, 2010, 2012, and 2014. MMWR Surveill. Summ..

[12] American Psychiatric Publishing (2013). Diagnostic and Statistical Manual of Mental Disorders: DSM-5^™^.

[13] Lord C., Risi S., Lambrecht L., Cook E.H., Leventhal B.L., DiLavore P.C., Pickles A., Rutter M. (2000). The autism diagnostic observation schedule-generic: A standard measure of social and communication deficits associated with the spectrum of autism. J. Autism Dev. Disord..

[14] Lord C., Rutter M., Le Couteur A. (1994). Autism Diagnostic Interview-Revised: A revised version of a diagnostic interview for caregivers of individuals with possible pervasive developmental disorders. J. Autism Dev. Disord..

[15] Yoon S.H., Choi J., Lee W.J., Do J.T. (2020). Genetic and Epigenetic Etiology Underlying Autism Spectrum Disorder. J. Clin. Med..

[16] Baron-Cohen S., Auyeung B., Norgaard-Pedersen B., Hougaard D.M., Abdallah M.W., Melgaard L., Cohen A.S., Chakrabarti B., Ruta L., Lombardo M.V. (2015). Elevated fetal steroidogenic activity in autism. Mol. Psychiatry.

[17] Pivovarciova A., Durdiakova J., Babinska K., Kubranska A., Vokalova L., Minarik G., Celec P., Murin M., Ostatnikova D. (2016). Testosterone and Androgen Receptor Sensitivity in Relation to Hyperactivity Symptoms in Boys with Autism Spectrum Disorders. PLoS ONE.

[18] Galiana-Simal A., Munoz-Martinez V., Calero-Bueno P., Vela-Romero M., Beato-Fernandez L. (2018). Towards a future molecular diagnosis of autism: Recent advances in biomarkers research from saliva samples. Int. J. Dev. Neurosci. Off. J. Int. Soc. Dev. Neurosci..

[19] Pfaffe T., Cooper-White J., Beyerlein P., Kostner K., Punyadeera C. (2011). Diagnostic potential of saliva: Current state and future applications. Clin. Chem..

[20] Roi A., Rusu L.C., Roi C.I., Luca R.E., Boia S., Munteanu R.I. (2019). A New Approach for the Diagnosis of Systemic and Oral Diseases Based on Salivary Biomolecules. Dis. Markers.

[21] Page M.J., McKenzie J.E., Bossuyt P.M., Boutron I., Hoffmann T.C., Mulrow C.D., Shamseer L., Tetzlaff J.M., Akl E.A., Brennan S.E. (2021). The PRISMA 2020 statement: An updated guideline for reporting systematic reviews. Syst. Rev..

[22] Said H.S., Suda W., Nakagome S., Chinen H., Oshima K., Kim S., Kimura R., Iraha A., Ishida H., Fujita J. (2014). Dysbiosis of salivary microbiota in inflammatory bowel disease and its association with oral immunological biomarkers. DNA Res. Int. J. Rapid Publ. Rep. Genes Genomes.

[23] Banasova L., Kamodyova N., Jansakova K., Tothova L., Stanko P., Turna J., Celec P. (2015). Salivary DNA and markers of oxidative stress in patients with chronic periodontitis. Clin. Oral Investig..

[24] Jansakova K., Escudier M., Tothova L., Proctor G. (2021). Salivary changes in oxidative stress related to inflammation in oral and gastrointestinal diseases. Oral Dis..

[25] Granger D.A., Kivlighan K.T., Fortunato C., Harmon A.G., Hibel L.C., Schwartz E.B., Whembolua G.L. (2007). Integration of salivary biomarkers into developmental and behaviorally-oriented research: Problems and solutions for collecting specimens. Physiol. Behav..

[26] Zimmermann L.K. (2008). A salivary collection method for young children. Psychophysiology.

[27] Wiener R.C., Wu B., Crout R., Wiener M., Plassman B., Kao E., McNeil D. (2010). Hyposalivation and xerostomia in dentate older adults. J. Am. Dent. Assoc..

[28] Jensen S.B., Vissink A. (2014). Salivary gland dysfunction and xerostomia in Sjogren’s syndrome. Oral Maxillofac. Surg. Clin. N. Am..

[29] Putnam S.K., Lopata C., Fox J.D., Thomeer M.L., Rodgers J.D., Volker M.A., Lee G.K., Neilans E.G., Werth J. (2012). Comparison of saliva collection methods in children with high-functioning autism spectrum disorders: Acceptability and recovery of cortisol. Child. Psychiatry Hum. Dev..

[30] Holm-Hansen C., Tong G., Davis C., Abrams W.R., Malamud D. (2004). Comparison of oral fluid collectors for use in a rapid point-of-care diagnostic device. Clin. Diagn. Lab. Immunol..

[31] Gomar-Vercher S., Simon-Soro A., Montiel-Company J.M., Almerich-Silla J.M., Mira A. (2018). Stimulated and unstimulated saliva samples have significantly different bacterial profiles. PLoS ONE.

[32] Okuma N., Saita M., Hoshi N., Soga T., Tomita M., Sugimoto M., Kimoto K. (2017). Effect of masticatory stimulation on the quantity and quality of saliva and the salivary metabolomic profile. PLoS ONE.

[33] Jasim H., Olausson P., Hedenberg-Magnusson B., Ernberg M., Ghafouri B. (2016). The proteomic profile of whole and glandular saliva in healthy pain-free subjects. Sci. Rep..

[34] Nunes L.A., Mussavira S., Bindhu O.S. (2015). Clinical and diagnostic utility of saliva as a non-invasive diagnostic fluid: A systematic review. Biochem. Med..

[35] Duarte D., Castro B., Pereira J.L., Marques J.F., Costa A.L., Gil A.M. (2020). Evaluation of Saliva Stability for NMR Metabolomics: Collection and Handling Protocols. Metabolites.

[36] Esser D., Alvarez-Llamas G., de Vries M.P., Weening D., Vonk R.J., Roelofsen H. (2008). Sample Stability and Protein Composition of Saliva: Implications for Its Use as a Diagnostic Fluid. Biomark. Insights.

[37] Takehara S., Yanagishita M., Podyma-Inoue K.A., Kawaguchi Y. (2013). Degradation of MUC7 and MUC5B in human saliva. PLoS ONE.

[38] Durdiakova J., Fabryova H., Koborova I., Ostatnikova D., Celec P. (2013). The effects of saliva collection, handling and storage on salivary testosterone measurement. Steroids.

[39] Bhattarai K.R., Kim H.R., Chae H.J. (2018). Compliance with Saliva Collection Protocol in Healthy Volunteers: Strategies for Managing Risk and Errors. Int. J. Med. Sci..

[40] Hicks S.D., Uhlig R., Afshari P., Williams J., Chroneos M., Tierney-Aves C., Wagner K., Middleton F.A. (2018). Oral microbiome activity in children with autism spectrum disorder. Autism Res. Off. J. Int. Soc. Autism Res..

[41] Kong X., Liu J., Cetinbas M., Sadreyev R., Koh M., Huang H., Adeseye A., He P., Zhu J., Russell H. (2019). New and Preliminary Evidence on Altered Oral and Gut Microbiota in Individuals with Autism Spectrum Disorder (ASD): Implications for ASD Diagnosis and Subtyping Based on Microbial Biomarkers. Nutrients.

[42] Qiao Y., Wu M., Feng Y., Zhou Z., Chen L., Chen F. (2018). Alterations of oral microbiota distinguish children with autism spectrum disorders from healthy controls. Sci. Rep..

[43] Pappa E., Vastardis H., Rahiotis C. (2020). Chair-side saliva diagnostic tests: An evaluation tool for xerostomia and caries risk assessment in children with type 1 diabetes. J. Dent..

[44] Farah R., Haraty H., Salame Z., Fares Y., Ojcius D.M., Said Sadier N. (2018). Salivary biomarkers for the diagnosis and monitoring of neurological diseases. Biomed. J..

[45] Blomqvist M., Bejerot S., Dahllof G. (2015). A cross-sectional study on oral health and dental care in intellectually able adults with autism spectrum disorder. BMC Oral Health.

[46] Kuter B., Guler N. (2019). Caries experience, oral disorders, oral hygiene practices and socio-demographic characteristics of autistic children. Eur. J. Paediatr. Dent..

[47] Qiao Y., Shi H., Wang H., Wang M., Chen F. (2020). Oral Health Status of Chinese Children with Autism Spectrum Disorders. Front. Psychiatry.

[48] Diab H.M., Motlaq S.S., Alsharare A., Alshammery A., Alshammery N., Khawja S.G., Shah A.H. (2016). Comparison of Gingival Health and Salivary Parameters among Autistic and Non-Autistic School Children in Riyadh. J. Clin. Diagn. Res..

[49] Kuter B., Uzel I. (2021). Evaluation of oral health status and oral disorders of children with autism spectrum disorders by gender. Arch. Pediatr..

[50] Morales-Chavez M.C., Villarroel-Dorrego M., Salas V. (2019). Salivary Factors Related to Caries in Children with Autism. J. Clin. Pediatr. Dent..

[51] Zhang Y., Lin L., Liu J., Shi L., Lu J. (2020). Dental Caries Status in Autistic Children: A Meta-analysis. J. Autism Dev. Disord..

[52] Leiva-García B., Planells E., del Pozo P.P., Molina-López J. (2019). Association between Feeding Problems and Oral Health Status in Children with Autism Spectrum Disorder. J. Autism Dev. Disord..

[53] Malhi P., Venkatesh L., Bharti B., Singhi P. (2017). Feeding Problems and Nutrient Intake in Children with and without Autism: A Comparative Study. Indian J. Pediatr..

[54] Ahearn W.H., Castine T., Nault K., Green G. (2001). An assessment of food acceptance in children with autism or pervasive developmental disorder-not otherwise specified. J. Autism Dev. Disord..

[55] Bolte E.R. (1998). Autism and Clostridium tetani. Med. Hypotheses.

[56] Finegold S.M., Molitoris D., Song Y., Liu C., Vaisanen M.L., Bolte E., McTeague M., Sandler R., Wexler H., Marlowe E.M. (2002). Gastrointestinal microflora studies in late-onset autism. Clin. Infect. Dis..

[57] Bhandary S., Hari N. (2017). Salivary biomarker levels and oral health status of children with autistic spectrum disorders: A comparative study. Eur. Arch. Paediatr. Dent..

[58] Braam W., Keijzer H., Struijker Boudier H., Didden R., Smits M., Curfs L. (2013). CYP1A2 polymorphisms in slow melatonin metabolisers: A possible relationship with autism spectrum disorder?. J. Intellect. Disabil. Res..

[59] Ostheim P., Tichy A., Sirak I., Davidkova M., Stastna M.M., Kultova G., Paunesku T., Woloschak G., Majewski M., Port M. (2020). Overcoming challenges in human saliva gene expression measurements. Sci. Rep..

[60] De Rubeis S., Buxbaum J.D. (2015). Genetics and genomics of autism spectrum disorder: Embracing complexity. Hum. Mol. Genet..

[61] Bralten J., van Hulzen K.J., Martens M.B., Galesloot T.E., Vasquez A.A., Kiemeney L.A., Buitelaar J.K., Muntjewerff J.W., Franke B., Poelmans G. (2018). Autism spectrum disorders and autistic traits share genetics and biology. Mol. Psychiatry.

[62] Guo H., Duyzend M.H., Coe B.P., Baker C., Hoekzema K., Gerdts J., Turner T.N., Zody M.C., Beighley J.S., Murali S.C. (2019). Genome sequencing identifies multiple deleterious variants in autism patients with more severe phenotypes. Genet. Med..

[63] Feliciano P., Zhou X., Astrovskaya I., Turner T.N., Wang T., Brueggeman L., Barnard R., Hsieh A., Snyder L.G., Muzny D.M. (2019). Exome sequencing of 457 autism families recruited online provides evidence for autism risk genes. NPJ Genom. Med..

[64] Jacquemont S., Coe B.P., Hersch M., Duyzend M.H., Krumm N., Bergmann S., Beckmann J.S., Rosenfeld J.A., Eichler E.E. (2014). A higher mutational burden in females supports a “female protective model” in neurodevelopmental disorders. Am. J. Hum. Genet..

[65] Bourgeron T. (2015). From the genetic architecture to synaptic plasticity in autism spectrum disorder. Nat. Rev. Neurosci..

[66] Hicks S.D., Ignacio C., Gentile K., Middleton F.A. (2016). Salivary miRNA profiles identify children with autism spectrum disorder, correlate with adaptive behavior, and implicate ASD candidate genes involved in neurodevelopment. BMC Pediatrics.

[67] Coolen M., Bally-Cuif L. (2009). MicroRNAs in brain development and physiology. Curr. Opin. Neurobiol..

[68] Hu Y., Ehli E.A., Boomsma D.I. (2017). MicroRNAs as biomarkers for psychiatric disorders with a focus on autism spectrum disorder: Current progress in genetic association studies, expression profiling, and translational research. Autism Res..

[69] Shen L., Lin Y., Sun Z., Yuan X., Chen L., Shen B. (2016). Knowledge-Guided Bioinformatics Model for Identifying Autism Spectrum Disorder Diagnostic MicroRNA Biomarkers. Sci. Rep..

[70] Konecna B., Radosinska J., Kemenyova P., Repiska G. (2020). Detection of disease-associated microRNAs—Application for autism spectrum disorders. Rev. Neurosci..

[71] Hicks S.D., Rajan A.T., Wagner K.E., Barns S., Carpenter R.L., Middleton F.A. (2018). Validation of a Salivary RNA Test for Childhood Autism Spectrum Disorder. Front. Genet..

[72] Wagner K.E., McCormick J.B., Barns S., Carney M., Middleton F.A., Hicks S.D. (2020). Parent Perspectives towards Genetic and Epigenetic Testing for Autism Spectrum Disorder. J. Autism Dev. Disord..

[73] Hicks S.D., Carpenter R.L., Wagner K.E., Pauley R., Barros M., Tierney-Aves C., Barns S., Greene C.D., Middleton F.A. (2020). Saliva MicroRNA Differentiates Children with Autism from Peers with Typical and Atypical Development. J. Am. Acad. Child. Adolesc. Psychiatry.

[74] Sehovic E., Spahic L., Smajlovic-Skenderagic L., Pistoljevic N., Dzanko E., Hajdarpasic A. (2020). Identification of developmental disorders including autism spectrum disorder using salivary miRNAs in children from Bosnia and Herzegovina. PLoS ONE.

[75] Levitskiy D., Confair A., Wagner K.E., DeVita S., Shea N., McKernan E.P., Kopec J., Russo N., Middleton F.A., Hicks S.D. (2021). Longitudinal stability of salivary microRNA biomarkers in children and adolescents with autism spectrum disorder. Res. Autism Spectr. Disord..

[76] Ragusa M., Santagati M., Mirabella F., Lauretta G., Cirnigliaro M., Brex D., Barbagallo C., Domini C.N., Gulisano M., Barone R. (2020). Potential Associations among Alteration of Salivary miRNAs, Saliva Microbiome Structure, and Cognitive Impairments in Autistic Children. Int. J. Mol. Sci..

[77] Wu Y.E., Parikshak N.N., Belgard T.G., Geschwind D.H. (2016). Genome-wide, integrative analysis implicates microRNA dysregulation in autism spectrum disorder. Nat. Neurosci..

[78] De Magistris L., Familiari V., Pascotto A., Sapone A., Frolli A., Iardino P., Carteni M., De Rosa M., Francavilla R., Riegler G. (2010). Alterations of the intestinal barrier in patients with autism spectrum disorders and in their first-degree relatives. J. Pediatr. Gastroenterol. Nutr..

[79] Goodwin M.S., Cowen M.A., Goodwin T.C. (1971). Malabsorption and cerebral dysfunction: A multivariate and comparative study of autistic children. J. Autism Child. Schizophr..

[80] D’Eufemia P., Celli M., Finocchiaro R., Pacifico L., Viozzi L., Zaccagnini M., Cardi E., Giardini O. (1996). Abnormal intestinal permeability in children with autism. Acta Paediatr..

[81] Sun Z., Cade J.R., Fregly M.J., Privette R.M. (1999). β-Casomorphin Induces Fos-Like Immunoreactivity in Discrete Brain Regions Relevant to Schizophrenia and Autism. Autism Int. J. Res. Pract..

[82] Van Sadelhoff J.H.J., Perez Pardo P., Wu J., Garssen J., van Bergenhenegouwen J., Hogenkamp A., Hartog A., Kraneveld A.D. (2019). The Gut-Immune-Brain Axis in Autism Spectrum Disorders; A Focus on Amino Acids. Front. Endocrinol..

[83] Benach J.L., Li E., McGovern M.M. (2012). A microbial association with autism. mBio.

[84] Jaber M.A. (2011). Dental caries experience, oral health status and treatment needs of dental patients with autism. J. Appl. Oral Sci..

[85] Jaber M.A., Sayyab M., Abu Fanas S.H. (2011). Oral health status and dental needs of autistic children and young adults. J. Investig. Clin. Dent..

[86] Krajmalnik-Brown R., Lozupone C., Kang D.W., Adams J.B. (2015). Gut bacteria in children with autism spectrum disorders: Challenges and promise of studying how a complex community influences a complex disease. Microb. Ecol. Health Dis..

[87] Galland L. (2014). The gut microbiome and the brain. J. Med. Food.

[88] Forsyth A., Raslan K., Lyashenko C., Bona S., Snow M., Khor B., Herrman E., Ortiz S., Choi D., Maier T. (2020). Children with autism spectrum disorder: Pilot studies examining the salivary microbiome and implications for gut metabolism and social behavior. Hum. Microbiome J..

[89] Ding H.T., Taur Y., Walkup J.T. (2017). Gut Microbiota and Autism: Key Concepts and Findings. J. Autism Dev. Disord..

[90] Kang V., Wagner G.C., Ming X. (2014). Gastrointestinal dysfunction in children with autism spectrum disorders. Autism Res. Off. J. Int. Soc. Autism Res..

[91] Malamud D. (2011). Saliva as a diagnostic fluid. Dent. Clin. N. Am..

[92] Alhajj M., Farhana A. (2021). Enzyme Linked Immunosorbent Assay. StatPearls 2021.

[93] Majewska M.D., Hill M., Urbanowicz E., Rok-Bujko P., Bienkowski P., Namyslowska I., Mierzejewski P. (2014). Marked elevation of adrenal steroids, especially androgens, in saliva of prepubertal autistic children. Eur. Child. Adolesc. Psychiatry.

[94] Hollocks M.J., Howlin P., Papadopoulos A.S., Khondoker M., Simonoff E. (2014). Differences in HPA-axis and heart rate responsiveness to psychosocial stress in children with autism spectrum disorders with and without co-morbid anxiety. Psychoneuroendocrinology.

[95] Bakker-Huvenaars M.J., Greven C.U., Herpers P., Wiegers E., Jansen A., van der Steen R., van Herwaarden A.E., Baanders A.N., Nijhof K.S., Scheepers F. (2020). Saliva oxytocin, cortisol, and testosterone levels in adolescent boys with autism spectrum disorder, oppositional defiant disorder/conduct disorder and typically developing individuals. Eur. Neuropsychopharmacol. J. Eur. Coll. Neuropsychopharmacol..

[96] Tomarken A.J., Han G.T., Corbett B.A. (2015). Temporal patterns, heterogeneity, and stability of diurnal cortisol rhythms in children with autism spectrum disorder. Psychoneuroendocrinology.

[97] Muscatello R.A., Corbett B.A. (2018). Comparing the effects of age, pubertal development, and symptom profile on cortisol rhythm in children and adolescents with autism spectrum disorder. Autism Res. Off. J. Int. Soc. Autism Res..

[98] Tordjman S., Anderson G.M., Kermarrec S., Bonnot O., Geoffray M.M., Brailly-Tabard S., Chaouch A., Colliot I., Trabado S., Bronsard G. (2014). Altered circadian patterns of salivary cortisol in low-functioning children and adolescents with autism. Psychoneuroendocrinology.

[99] Corbett B.A., Mendoza S., Abdullah M., Wegelin J.A., Levine S. (2006). Cortisol circadian rhythms and response to stress in children with autism. Psychoneuroendocrinology.

[100] Jamnadass E.S., Keelan J.A., Hollier L.P., Hickey M., Maybery M.T., Whitehouse A.J. (2015). The perinatal androgen to estrogen ratio and autistic-like traits in the general population: A longitudinal pregnancy cohort study. J. Neurodev. Disord..

[101] Auyeung B., Taylor K., Hackett G., Baron-Cohen S. (2010). Foetal testosterone and autistic traits in 18 to 24-month-old children. Mol. Autism.

[102] Procyshyn T.L., Lombardo M.V., Lai M.C., Auyeung B., Crockford S.K., Deakin J., Soubramanian S., Sule A., Baron-Cohen S., Bethlehem R.A.I. (2020). Effects of oxytocin administration on salivary sex hormone levels in autistic and neurotypical women. Mol. Autism.

[103] Fujioka T., Fujisawa T.X., Inohara K., Okamoto Y., Matsumura Y., Tsuchiya K.J., Katayama T., Munesue T., Tomoda A., Wada Y. (2020). Attenuated relationship between salivary oxytocin levels and attention to social information in adolescents and adults with autism spectrum disorder: A comparative study. Ann. Gen. Psychiatry.

[104] Groschl M., Wagner R., Rauh M., Dorr H.G. (2001). Stability of salivary steroids: The influences of storage, food and dental care. Steroids.

[105] Garde A.H., Hansen A.M. (2005). Long-term stability of salivary cortisol. Scand. J. Clin. Lab. Investig..

[106] Skoluda N., La Marca R., Gollwitzer M., Muller A., Limm H., Marten-Mittag B., Gundel H., Angerer P., Nater U.M. (2017). Long-term stability of diurnal salivary cortisol and alpha-amylase secretion patterns. Physiol. Behav..

[107] Zhu C., Yuan C., Ren Q., Wei F., Yu S., Sun X., Zheng S. (2021). Comparative analysis of the effects of collection methods on salivary steroids. BMC Oral Health.

[108] Spratt E.G., Nicholas J.S., Brady K.T., Carpenter L.A., Hatcher C.R., Meekins K.A., Furlanetto R.W., Charles J.M. (2012). Enhanced cortisol response to stress in children in autism. J. Autism Dev. Disord..

[109] Kidd S.A., Corbett B.A., Granger D.A., Boyce W.T., Anders T.F., Tager I.B. (2012). Daytime secretion of salivary cortisol and alpha-amylase in preschool-aged children with autism and typically developing children. J. Autism Dev. Disord..

[110] Corbett B.A., Mendoza S., Wegelin J.A., Carmean V., Levine S. (2008). Variable cortisol circadian rhythms in children with autism and anticipatory stress. J. Psychiatry Neurosci. Jpn..

[111] Abdulla A.M., Hegde A.M. (2015). Salivary Cortisol Levels and its Implication on Behavior In Children with Autism during Dental Treatment. J. Clin. Pediatric Dent..

[112] Ander S.E., Diamond M.S., Coyne C.B. (2019). Immune responses at the maternal-fetal interface. Sci. Immunol..

[113] Boulanger-Bertolus J., Pancaro C., Mashour G.A. (2018). Increasing Role of Maternal Immune Activation in Neurodevelopmental Disorders. Front. Behav. Neurosci..

[114] Jash S., Sharma S. (2021). In utero immune programming of autism spectrum disorder (ASD). Hum. Immunol..

[115] Saghazadeh A., Ataeinia B., Keynejad K., Abdolalizadeh A., Hirbod-Mobarakeh A., Rezaei N. (2019). A meta-analysis of pro-inflammatory cytokines in autism spectrum disorders: Effects of age, gender, and latitude. J. Psychiatr. Res..

[116] Loo J.A., Yan W., Ramachandran P., Wong D.T. (2010). Comparative human salivary and plasma proteomes. J. Dent. Res..

[117] Campanati A., Martina E., Diotallevi F., Radi G., Marani A., Sartini D., Emanuelli M., Kontochristopoulos G., Rigopoulos D., Gregoriou S. (2021). Saliva Proteomics as Fluid Signature of Inflammatory and Immune-Mediated Skin Diseases. Int. J. Mol. Sci..

[118] Castagnola M., Messana I., Inzitari R., Fanali C., Cabras T., Morelli A., Pecoraro A.M., Neri G., Torrioli M.G., Gurrieri F. (2008). Hypo-phosphorylation of salivary peptidome as a clue to the molecular pathogenesis of autism spectrum disorders. J. Proteome Res..

[119] Ngounou Wetie A.G., Wormwood K.L., Russell S., Ryan J.P., Darie C.C., Woods A.G. (2015). A Pilot Proteomic Analysis of Salivary Biomarkers in Autism Spectrum Disorder. Autism Res. Off. J. Int. Soc. Autism Res..

[120] Ngounou Wetie A.G., Wormwood K.L., Charette L., Ryan J.P., Woods A.G., Darie C.C. (2015). Comparative two-dimensional polyacrylamide gel electrophoresis of the salivary proteome of children with autism spectrum disorder. J. Cell Mol. Med..

[121] Samborska-Mazur J., Kostiukow A., Miechowicz I., Sikorska D., Rutkowski R., Wyganowska-Swiatkowska M., Blochowiak K. (2020). Salivary Cytokine Profile as a Possible Predictor of Autism Spectrum Disorder. J. Clin. Med..

[122] Wang K., Wang X., Zheng S., Niu Y., Zheng W., Qin X., Li Z., Luo J., Jiang W., Zhou X. (2018). iTRAQ-based quantitative analysis of age-specific variations in salivary proteome of caries-susceptible individuals. J. Transl. Med..

[123] Kobayashi H., Song C., Ikei H., Park B.J., Kagawa T., Miyazaki Y. (2017). Diurnal Changes in Distribution Characteristics of Salivary Cortisol and Immunoglobulin A Concentrations. Int. J. Environ. Res. Public Health.

[124] Chen L., Shi X.J., Liu H., Mao X., Gui L.N., Wang H., Cheng Y. (2021). Oxidative stress marker aberrations in children with autism spectrum disorder: A systematic review and meta-analysis of 87 studies (N = 9109). Transl. Psychiatry.

[125] Rai K., Hegde A.M., Jose N. (2012). Salivary antioxidants and oral health in children with autism. Arch. Oral Biol..

[126] Fahad A., Jafer N. (2017). Salivary Physicochemical Characteristics in Relation to Oral Health Status among Institutionalized Autistic Adolescents in Baghdad/Iraq. J. Baghdad Coll. Dent..

[127] Karamouzi A., Kovachev D., Karamouzis I., Antoniadou-Hitoglou M., Tsikoulas I., Aggelopoulou-Sakadami N. (2007). Saliva Levels of 15-F 2t -Isoprostane as Biomarker of Lipid Peroxidation in Autistic Children. Eur. J. Inflamm..

[128] Lettrichova I., Tothova L., Hodosy J., Behuliak M., Celec P. (2016). Variability of salivary markers of oxidative stress and antioxidant status in young healthy individuals. Redox Rep. Commun. Free Radic. Res..

[129] Demirci E., Guler Y., Ozmen S., Canpolat M., Kumandas S. (2019). Levels of Salivary Sialic Acid in Children with Autism Spectrum Disorder; Could It Be Related to Stereotypes and Hyperactivity?. Clin. Psychopharmacol. Neurosci. Off. Sci. J. Korean Coll. Neuropsychopharmacol..

[130] Kubala E., Strzelecka P., Grzegocka M., Lietz-Kijak D., Gronwald H., Skomro P., Kijak E. (2018). A Review of Selected Studies That Determine the Physical and Chemical Properties of Saliva in the Field of Dental Treatment. Biomed. Res. Int..

[131] Loke C., Lee J., Sander S., Mei L., Farella M. (2016). Factors affecting intra-oral pH—A review. J. Oral Rehabil..

[132] Bassoukou I.H., Nicolau J., dos Santos M.T. (2009). Saliva flow rate, buffer capacity, and pH of autistic individuals. Clin. Oral Investig..

